# Supplementary material for: Analysis of PPARγ Signaling Activity in Psoriasis
Source: Int J Mol Sci. 2021 Aug 10;22(16):8603. doi: 10.3390/ijms22168603 (PMC8395241; doi:10.3390/ijms22168603)
Supplement: Supplementary file 1 [file ijms-22-08603-s001.zip › Supplemental materials_Analysis of PPARg signaling activity in psoriasis/Pathway models/Models images and html files/PPARG inhibits phenotypes assotiated with psoriasis/186550.html]

platelet activation


# Cell Process platelet activation

|  |  |
| --- | --- |
| URN | urn:agi-gocellproc:0030168 |
| Total Entities | 0 |
| Connectivity | 2364 |
| Name | platelet activation |

---

|  |  |
| --- | --- |
| Pathway | Scavenger Receptors in Platelet Activation |
|  | Proplatelet Maturation |
|  | Bradykinin Effects in Inflammation |
|  | Coagulation Cascade |
|  | Trophoblast Damage in Infertility (Hypothesis) |
|  | Hereditary Thrombophilia and Pregnancy Complications |
|  | Thrombophilia Hereditary |
|  | Plasmin Role in Glomerulonephritis |
|  | PPARG inhibits phenotypes assotiated with psoriasis |
|  | efects selected |

---

|  |  |
| --- | --- |
| MedScan ID | 4130168 |

---

|  |  |
| --- | --- |
| Alias | platelets activation |
|  | activated platelets |
|  | platelet activation |
|  | activated platelet |
|  | thrombocyte stimulation |
|  | stimulation of platelet |
|  | platelet activations |
|  | blood platelet activation |
|  | platelet stimulation |
|  | activation of platelet |
|  | blood platelet stimulation |
|  | thrombocyte activations |
|  | thrombocyte activation |

---

|  |  |
| --- | --- |
| GO ID | 0030168 |

---
